# Changes of tau profiles in brains of the hamsters infected with scrapie strains 263 K or 139 A possibly associated with the alteration of phosphate kinases

**DOI:** 10.1186/1471-2334-10-86

**Published:** 2010-04-01

**Authors:** Gui-Rong Wang, Song Shi, Chen Gao, Bao-Yun Zhang, Chan Tian, Chen-Fang Dong, Rui-Min Zhou, Xiao-Li Li, Cao Chen, Jun Han, Xiao-Ping Dong

**Affiliations:** 1 State Key Laboratory for Infectious Disease Prevention and Control, National Institute for Viral Disease Control and Prevention, Chinese Center for Disease Control and Prevention, Ying-Xin Rd 100, Beijing 100052, PR China

## Abstract

**Background:**

Phospho-tau deposition has been described in a rare genetic human prion disease, Gerstmann-Sträussler-Scheinker syndrome, but is not common neuropathological picture for other human and animal transmissible spongiform encephalopathies (TSEs). This study investigated the possible changes of tau and phosphorylated tau (p-tau, at Ser396, Ser404, and Ser202/Thr205) in scrapie experimental animals.

**Methods:**

The profiles of tau and p-tau (p-tau, at Ser396, Ser404, and Ser202/Thr205) in the brain tissues of agents 263K- or 139A-infected hamsters were evaluated by Western blots and real-time PCR. Meanwhile, the transcriptional and expressive levels of GSK3β and CDK5 in the brains were tested.

**Results:**

The contents of total tau and p-tau at Ser202/Thr205 increased, but p-tau at Ser396 and Ser404 decreased at the terminal stages, regardless of scrapie strains. Transcriptional levels of two tau isoforms were also increased. Additionally, it showed higher CDK5, but lower GSK3β transcriptional and expressive levels in the brains of scrapie-infected animals. Analysis of brain samples collected from different times after inoculated with agent 263 K revealed that the changes of tau profiles and phosphate kinases were time-relative events.

**Conclusion:**

These data suggest that changes of profiles of p-tau at Ser396, Ser404 and Ser202/Thr205 are illness-correlative phenomena in TSEs, which may arise of the alteration of phosphate kinases. Alteration of tau, p-tau (Ser396, Ser404, and Ser202/Thr205), GSK3β and CDK5 were either intermediate or consequent events in TSE pathogenesis and proposed the potential linkage of these bioactive proteins with the pathogenesis of prion diseases.

## Background

Prion diseases, also known as transmissible spongiform encephalopathies (TSEs) are a group of fatal neurodegenerative diseases of animals and humans causing severe spongiform degeneration and neuronal loss in central nervous system (CNS). This relatively diverse group includes bovine spongiform encephalopathy in cattle (BSE), scrapie in sheep and goat, chronic wasting disease (CWD) in deer and elk, and kuru, Creutzfeldt-Jakob disease (CJD), fatal familial insomnia (FFI) and Gerstmann-Sträussler-Scheinker (GSS) disease in humans [1,2]. Clinically, prion diseases can exhibit sporadic, inherited, or infectious presentations. Regardless of the primary etiology, the central event in the pathogenesis is the conformational conversion from the cellular prion protein (PrP^C^) into its insoluble and protease-resistant forms (termed PrP^Sc^) that accumulate in CNS [1].

The microtubule-associated protein tau, is a group of molecular mass of 45-66 kDa proteins encoded by alternative splicing of a single gene [3]. There are six predominant tau isoforms in human brain containing 352-441 amino acids [4]. Hyperphosphorylated tau aggregated into paired helical filaments, which is the main component of neurofibrillary tangles found in the brains of Alzheimer's disease (AD) patients [5]. The main aberrantly hyperphosphorylated sites on tau include the phospho-sites Ser-202/Thr-205 (AT8 site), Ser-214 and/or Ser-212 (AT100 site), Thr-231 and/or Ser-235 (TG3 site), and Ser-396/Ser-404 (PHF-1 site) [6]. Precise regulation of phosphorylation of tau is probably important for its normal cellular functions and aberrant tau phosphorylation is believed to disrupt cellular processes such as axonal transport [7].

The normal phosphorylation state of tau is balanced by antagonistic kinase and phosphatase activity. Some kinases and phosphatases have been implicated in the abnormal hyperphosphorylation of tau [7]. The proline-directed Serine/Threonine kinases, cyclin-dependent kinase 5 (CDK5) and glycogen synthase kinase 3β (GSK3β) have been identified as prime candidates mediating aberrant tau phosphorylation at disease-associated sites [8]. CDK5 co-localizes with filamentous tau deposits and has increased activity in several tauopathies, such as AD [9]. GSK3β generates disease associated phospho-epitopes on tau [10] and co-localizes with aggregates of hyperphosphorylated tau [11]. Obtained data show that apoptotic neuronal death caused by PrP_106-126 _is in part due to CDK5 deregulation, causing tau hyperhosphorylation at Ser202/Thr205 and apoptotic death. The inhibitors of CDK5 and calpain reverted tau hyperphosphorylation and prevented neuronal death caused by PrP_106-126 _[12]. Over-activation of CDK5 in young transgenic animals does not induce tau hyperphosphorylation at sites recognized by the antibodies AT8, AT100, PHF-1 and TG3, while increased GSK3β activity coincides with tau hyperphosphorylation at the AT8 and PHF-1 sites, which reveales the role of GSK3β as a key mediator of tau hyperphosphorylation, whereas CDK5 acts as a modulator of tau hyperphosphorylation via the inhibitory regulation of GSK3β [7]. Both GSK3β and CDK5 can phosphorylate tau at many positions, e.g. Thr181, Ser199, Ser202, Thr205, Thr212, Thr217, Ser396, and Ser404. However, GSK3β seems to prefer phosphorylating tau at its microtubule binding domains in the C-terminus, while CDK5 tends to target the N-terminus [13].

Human prion diseases share some clinical and neuropathological features with AD. Both are dementia disorders, whose pathogeneses are related to the deposition of abnormal proteins in the extracellular space of the brain and this change is considered to have a crucial role in causing the degeneration and death of neuronal cells. On the other hand, the two diseases have distinct natural processes. CJD is a unique transmissible encephalopathy and the clinical course is much shorter than AD. In sporadic CJD, PrP^Sc ^builds up in the gray matter mostly in diffuse form and tau pathology is not considered part of the neuropathological picture [14]. Although there are some reports about Alzheimer-type changes in CJD patients [15,16], these cases are elder patients, in which neurofibrillary changes are associated with Aβ deposits, likely representing the co-occurrence of CJD with AD or age-related phenomena. In the brain tissues of GSS, a familial prion disease caused by specific mutations of the PrP gene (PRNP), abundant hyperphosphorylated tau (p-tau) depositions have been documented [17]. To date, phospho-tau-related changes have been rarely reported in patients with vCJD [18,19], but one study describes that phospho-tau often clusters around PrP amyloid deposits in the brains of five vCJD patients and the mouse models of vCJD [20]. Besides, hyperphosphorylation of tau protein has been previously reported in bovine PrP transgenic mice infected with BSE [21].

In the present study, the relative quantity of total tau protein and its phosphorylated status at Ser396, Ser404, and Ser202/Thr205 in the brain tissues of the hamsters infected with scrapie agent 263 K or 139 A at their clinical terminal stages were evaluated. Western blots revealed that the amounts of total tau and p-tau at Ser202/Thr205 increased, but the levels of p-tau at Ser396 and Ser404 decreased. Transcription of two tau isoforms was also increased. In parallel, the expression and transcription of GSK3β were depressed, while those of CDK5 were increased in the brains of scrapie-infected animals.

## Methods

### Animal brain samples infected with scrapie agents

Five Chinese golden hamsters inoculated intracerebrally with hamster-adapted scrapie agent 263 K and five hamsters inoculated intracerebrally with mouse-adapted scrapie agent 139 A were included in this study. Previously studies confirmed that the incubation time of 263K-infected hamsters was 79.1 ± 8.6 days [22], while that of 139A-infected hamsters was 395 ± 8.5 days [23]. The brains were removed surgically within 8 hrs after natural death or at the moribund stage. The brains were immediately dissected, then frozen and stored at -80°C until use. Besides, two brain samples of the agent 263K-infected hamsters at the 20^th^, 40^th^, 50^th^, 60^th ^and 70th days after inoculation were collected. Besides, eight normal hamsters were enrolled as controls.

### Preparation of brain tissue samples

Brain homogenates of the hamsters were prepared described previously [24], with slight modification. Brain samples from the scrapie-infected and healthy hamsters were washed in TBS (10 mM Tris HCl, 133 mM NaCl, pH 7.4) for three times, and then 10% (W/V) brain homogenates were prepared in lysis buffer (100 mM NaCl, 10 mM EDTA, 0.5% Nonidet P-40, 0.5% sodium deoxycholate, 10 mM Tris, pH 7.5) containing a mixture of protease inhibitors. The tissue debris was removed by low-speed centrifugation at 2,000 g for 10 min and the supernatants were used for further experiments.

### Protease Resistance Assay

For detection of PrP^Sc ^in brain tissues, all tested brain homogenates were digested with a final concentration of 50 μg/ml proteinase K (PK) at 37°C for 60 min prior to Western blot, digestion was terminated by addition of PMSF to a final concentration of 5 mM and boiling in SDS-PAGE sample buffer.

### Antibodies

Anti-tau monoclonal antibody (mAb) tau13, and anti-β-actin mAb were purchased from Santa Cruz Biotechnology. Anti-PrP mAb 3F4 was purchased from Dako. Anti-p-tau (Ser396) polyclonal antibody (pAb) TAU [pS396], Anti-p-tau (Ser404) pAb TAU [pS404], Anti-GSK3β mAb, and Anti-CDK5 mAb were purchased from Biosource. Anti-p-tau (Ser202/Thr205) mAb AT8 was purchased from Pierce Endogen.

### Western blot analysis

Samples were separated by 15% SDS-PAGE and electroblotted onto a nitrocellulose membrane using a semi-dry blotting system (Bio-rad). Membranes were blocked with 5% (w/v) non-fat milk powder (NFMP) in 1 × Tris-buffered saline containing 0.1% Tween 20 (NFMP-TBST) at room temperature for 1 h and probed with mAb 3F4 (1:5000), mAb tau13 (1:4000), pAb TAU [pS396] (1:1000), pAb TAU [pS404] (1:1000), mAb AT8 (1:4000), anti-GSK3β mAb (1:4000), Anti-CDK5 mAb (1:4000) and anti-β-actin mAb (1:2000) at 4°C overnight, respectively. After washing three times with TBST, blots were incubated in HRP-conjugated secondary antibody solutions, 1:5,000-diluted HRP-conjugated goat anti-rabbit IgG or goat anti-mouse IgG (Santa Cruz, CA, USA), at room temperature for 2 h. Blots were developed using Enhanced ChemoLuminescence system (ECL, Amersham Life Sciences, Buckinghamshire, UK) and visualized on autoradiography films.

### Real Time Reverse Transcription PCR

Total RNA from the brain of normal and scrapie-infected hamsters were extracted using the TRIzol (Invitrogen) reagent according to the manufacturer's instructions. Reverse transcription was performed using SuperScript™ III First-Strand Synthesis System (Invitrogen). Briefly, 2 μg of total RNA was mixed with 200 U of MMLV reverse transcriptase and 50 pM oligo (dT20) in a volume of 20 μl. The mixtures were maintained at 50°C for 50 min and inactivated by heating at 85°C for 5 min. To remove the RNA from the cDNA, 1 μl *E. coli *RNase H was added to the mixture and incubated at 37°C for 20 min. Aliquots (2 μl) of RT reaction products were amplified by PCR. The primers used were designed and synthesized according to the published literatures. Since no respective hamster's data available in GenBank, the primers were based on the individual sequences of mouse, including the primers for tau, Tau2-sence 5'-TCG CCA GGA GTT TGA CAC AAT GGA-3', Tau4-sence, 5'-CCA CAC GGA GAT CCC AGA AGG AAT TA-3' and Tau- antisence, 5'-ATT TCC TGT CCT GTC TTT GCT GGC-3' [7]; Tau6-sence 5'- TAC ACC ATG CAC CAA GAC CA-3' and Tau6-antisence 5'-GTC TCC AAT GCC TGC TTC TT-3 [25]; GSK3β-sence 5'-CTA AGG ATT CGT CAG GAA CAG-3' and GSK3β-antisence 5'-TTG AGT GGT GAA GTT GAA GAG-3 [26]; CDK5-sence 5'-ACTGTGTTCAAGGCTAAAAACC-3' and CDK5-antisence 5'-CAATTTCAACTCCCCATTCC-3' [27]; β-actin-sence 5'-CTA CAA TGA GCT GCG TGT GGC-3' and β-actin-antisence 5'-CAG GTC CAG ACG CAG GAT GGC-3' [28]. Real time-PCR was performed using a supermix containing the fluorescent dye SYBR green (Applied Biosystems) as described previously [29], briefly, 1 μl of reverse transcription product, 12.5 μl of 2× SYBR Green PCR Master Mix, 1 μl of each primer (10 μM) and 9.5 μl H_2_O were mixed in a total volume of 25 μl. PCR was performed on a 7500 Fast Real-Time PCR System (Applied Biosystems), under following condition 94°C for 15 s, 55°C for 30 s, and 72°C for 30 s, total 40 cycles. The increases in fluorescence were collected and the expressive level of each specific mRNA was determined relative to that of the individual β-actin. All real time-PCR reactions were performed in triplicate.

### Quantitative and statistical analysis

Quantitative analysis of immunoblot images was carried out using Image Total Tech software (Pharmacia). Briefly, the image of immunoblot was scanned with Typhoon (Pharmacia) and digitalized, saved in TIF format. The values of each target blot were evaluated. All data are presented as the mean ± SD. Student's unpaired t test for comparison of means was used to compare groups. Differences were considered significant at a P value < 0.05.

### Ethical approval

This study was approved by the Ethical Committee of National Institute for Viral Disease Prevention and Control, China CDC.

## Results

### Increase of total tau in the brain tissues of the scrapie-infected hamsters

**Figure 1:**
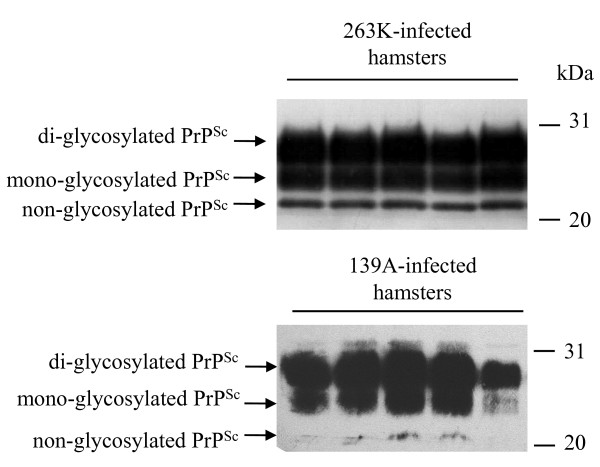
**Western blot analysis of PrP^Sc ^in the brain tissues of scrapie agents 263 K- and 139A-infected hamsters collected at the moribund stage**. For detection of PrP^Sc^, all tested brain homogenates were digested with a final concentration of 50 μg/ml PK at 37°C for 60 min. Same amounts of individual brain homogenate were loaded in 15% SDS-PAGE. The scrapie agents were shown on the top of the graphs. PrP^Sc ^specific immunoblots (non-, mono- and di-glycosylated PrP isoforms) were indicated by arrows on the left and molecular mass markers were indicated to the right.

To address the presences of PrP^Sc ^in the brain tissues of scrapie-infected animals, 10% brain homogenates of five strain 263K- and five 139A-infected hamsters were digested by PK and analyzed with PrP specific Western blots. Three PK-resistant PrP bands were identified in all infected samples, which moved at the position from Mr. 21 to 27 kDa (Figure 1), representing di-, mono- and aglycosal forms.

**Figure 2:**
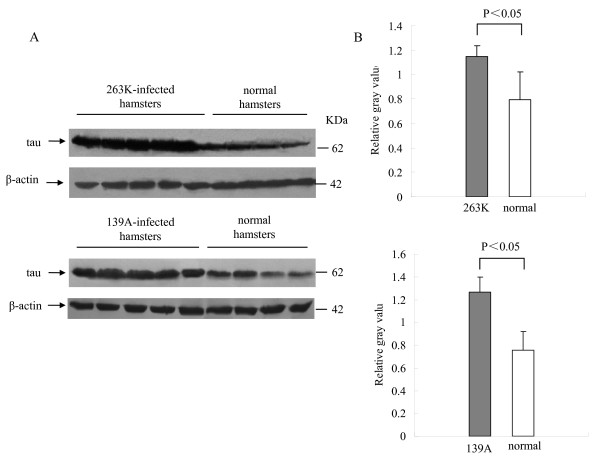
**Comparative analysis of the level of tau protein in the brain tissues of normal, scrapie agent 263K- and 139A-infected hamsters collected at the moribund stage**. A. Western blots. Same amounts of individual brain homogenate were loaded in 12% SDS-PAGE. The scrapie agents as well as normal control were shown on the top of the graphs. Tau specific immunoblots were indicated by arrows on the left and molecular mass markers were indicated to the right. B. Quantitative analyses of each gray numerical value of tau vs that of β-actin. The average values were calculated from five individual infected hamsters or four individual normal hamsters and presented as mean ± SD. Statistical differences compared with normal controls were illustrated as *P *< 0.05 and *P *< 0.01.

To assess the possible changes of tau in the brains of scrapie experimental hamsters at the terminal stage, the amounts of tau protein in brains were evaluated by Western blots with a tau-specific mAb, which was confirmed not to be influenced by the process of tau phosphorylation. At the experimental condition, tau-specific signals at the position of 63 kDa were detected. Compared with the healthy hamsters at similar ages, the signal intensities in agent 263K- or 139A-infected hamsters were obviously higher. Meanwhile, the levels of β-actin in the brain tissues were comparable between the infected and healthy groups (Figure 2A). To obtain more precise data, the gray value of tau signal in each sample was equilibrated with the individual value of β-actin. It showed that the mean relative quantities of tau protein in the brains of 263K- or 139A-infected hamsters were significantly higher than those of the healthy ones (Figure 2B, P < 0.05).

### More active transcription of tau mRNA in the brain tissues of the infected hamsters

**Figure 3:**
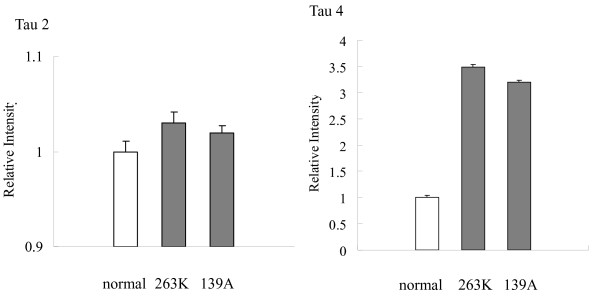
**Comparative analysis of the mRNA transcription of Tau2 and Tau4 in the brain tissues of normal and scrapie-infected hamsters collected at the moribund stage by real time PCR**. The transcription level of each specific mRNA was determined relative to that of the individual β-actin. The relative intensity of each gene from scrapie-infected hamsters was relative to that of respective gene from normal hamsters that was set to 1. Data are representative of three independent experiments.

Tau is encoded via the transcription of a single gene by alternative splicing, resulting in six isoforms [3]. To assess the transcription of tau mRNA in brains, several specific primer pairs targeting the tau isoforms Tau-2, Tau-4 and Tau-6 were designed based on the mouse tau sequences. RT-PCR assays using extracted RNA from normal and scrapie-infected hamsters as the templates revealed a 187 bp product with Tau-2-specific primers and a 127 bp fragment with Tau-4-specific primers, possibly representing the tau isoform of 372 amino acid residues and isoform of 432 amino acid residues, while the primers for Tau-6 failed to produce positive product (data not shown). To evaluate the transcriptional levels of Tau-2 and Tau-4 in the brain of each animal, real time-PCR reactions were performed. In addition, the transcription of the housekeeping gene encoding β-actin was also evaluated as an internal control. After equilibrated with Ct value of respective β-actin, the mean Ct values of Tau-2 and Tau-4 of each hamster were calculated from the data of three independent reactions. Analysis of the relative intensities of various groups showed that the levels of Tau-2 transcript in the 263K- and 139A-infected hamsters were slightly increased, but no significant difference was identified between the infected and the normal controls (Figure 3). The level of Tau-4 mRNA was remarkably increased in the brain tissues of 263K-infected (3.49-fold) and 139A-infected hamsters (3.20-fold). It highlights that the transcription of Tau-4 in the brains of scrapie-infected hamsters is more active at the terminal stage.

### Changes of phosphorylated tau profiles in the brain tissues of the scrapie-infected hamsters

**Figure 4:**
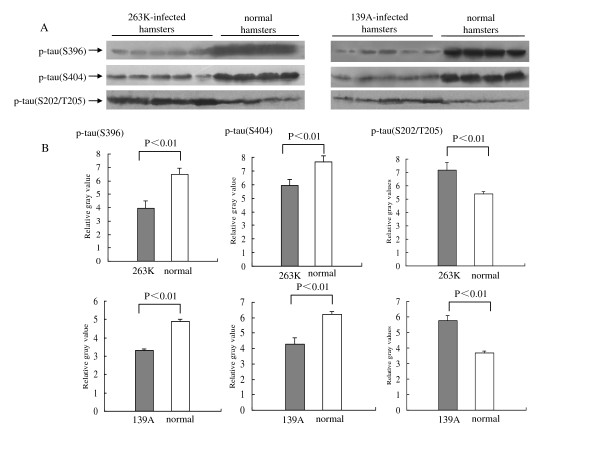
**Analysis of the levels of p-tau (Ser396), p-tau (Ser404) and p-tau (Ser202/Thr205) in the brain tissues of normal and scrapie-infected hamsters collected at the moribund stage**. A. Western blots. Same amounts of individual brain homogenate were loaded in 12% SDS-PAGE. The scrapie agents and normal controls were shown on the top of the graphs. Various specific immunoblots were indicated by arrows on the left. B. Quantitative analysis of each gray numerical value of p-tau (Ser396), p-tau (Ser404) and p-tau (Ser202/Thr205) vs that of β-actin. The average values were calculated from five individual infected hamsters or four individual normal hamsters and presented as mean ± SD. Statistical differences compared with controls were illustrated as *P *< 0.05 and *P *< 0.01.

To see the difference in the patterns of tau phosphorylation, all brain homogenates were screened by the Western blots with various p-tau-specific antibodies, including pAb TAU [pS396], pAb TAU [pS404] and mAb AT8, which recognize the tau proteins phosphorylated at Serine 396, Serine 404 and Serine 202/Thr 205, respectively. Antibody TAU [pS396] and TAU [pS404] revealed the same reactive profiles that the signal intensities in the hamsters infected with agent 263 K or 139 A were obviously lower than that of normal ones (Figure 4A). Scanning the image of each blots identified the mean relative quantities of p-tau (Ser396 and Ser404) in the infected hamsters were remarkably lower than that in the control group (P < 0.001, Figure 3).

A 63-kDa p-tau (Ser202/Thr205)-specific immunoblot was detected in the brain homogenates by the Western blot with mAb AT8. Contrast to the situation of p-tau (Ser396 and Ser404), mAb AT8 revealed the different pattern that the level of p-tau (Ser202/Thr205) in the brains infected with agent 263 K or 139 A was clearly stronger than that of the normal controls (Figure 4A). The mean relative quantities of p-tau (Ser202/Thr205) in the infected hamster brains were significantly higher than that in the normal controls (P < 0.001, Figure 4B). These data imply that the phosphorylating profiles of tau proteins in the brains of scrapie-infected hamsters changed at their clinically terminal stage.

### Lower level of GSK3β and higher level of CDK5 in the brain tissues of the scrapie-infected hamsters

**Figure 5:**
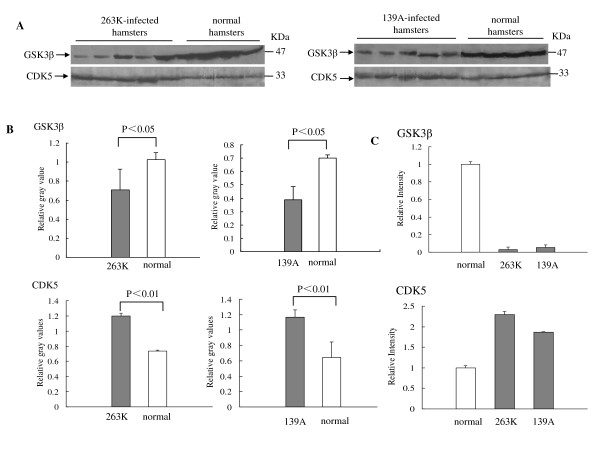
**Analysis of GSK3β and CDK5 in the brain tissues of normal and scrapie-infected hamsters collected at the moribund stage**. A. Western blots. Same amounts of individual brain homogenate were loaded in 12% SDS-PAGE. The scrapie agents and normal controls were shown on the top of the graphs. Various specific immunoblots were indicated by arrows on the left. B. Quantitative analysis of each gray numerical value of GSK3β and CDK5 vs that of β-actin. The average values were calculated from five individual infected hamsters or four individual normal hamsters and presented as mean ± SD. Statistical differences compared with controls were illustrated as *P *< 0.05 and *P *< 0.01. C. Real time PCR analysis of the mRNA transcription of GSK3β and CDK5. The transcription level of each specific mRNA was determined relative to that of the individual β-actin. The relative intensity of each gene from scrapie-infected hamsters was relative to that of respective gene from normal hamsters that was set to 1. Data are representative of three independent experiments.

GSK3β activity is believed to coincide with tau hyperphosphorylation at the AT8 and PHF-1 sites [7]. To test the possible changes of GSK3β in the central nerve tissues of animals with TSE, the expressive and transcriptional levels of GSK3β in the brains of normal and scrapie-infected hamsters were evaluated by GSK3β-specific Western blot and real-time PCR. Western blots identified that the GSK3β signals in 263K- or 139A-infected hamsters were obviously weaker than that in normal controls (Figure 5A). Analysis of the immunoblots of GSK3β showed that the relative quantities of GSK3β signals in the groups of 263K- or 139A-infected hamsters were clearly lower (Figure 5B, P < 0.05). Real-time PCR for the transcription of GSK3β also revealed the similar pattern, that the levels of GSK3β transcript were downregulated by 0.03-fold in the brain of 263K-infected hamsters and 0.06-fold in 139A-infected hamsters. To see the status of CDK5 in brains of scrapie experimental hamsters, same brain homogenates from 263K- or 139A-infected hamsters and healthy ones were employed into CDK5-specific Western blots and real-time PCR. Western blots showed much stronger CDK5 signals in all tested brains of scrapie experimental hamsters, whose mean relative intensities were clearly higher than that of healthy ones (P < 0.001, Figure 5B). Real-time PCR for CKD5 demonstrated that the amounts of CDK5 transcript were remarkably upregulated in the brain tissues of 263K-infected (2.3-fold) and 139A-infected hamsters (1.87-fold) (Figure 5C).

### Changes of tau profiles and phosphate kinases are time-relative events

**Figure 6:**
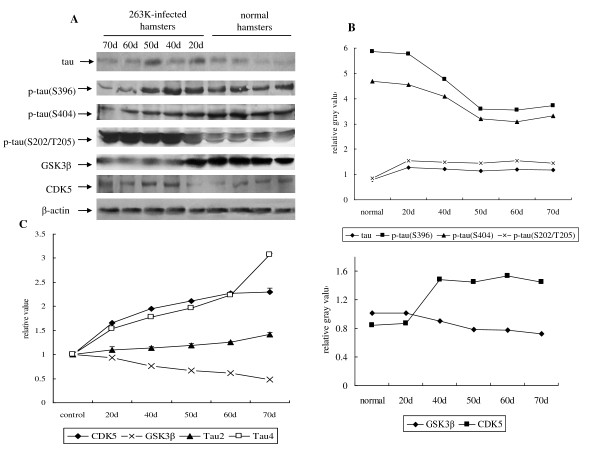
**Dynamic analyses of tau, p-tau (Ser396), p-tau (Ser404), p-tau (Ser202/Thr205), GSK3β and CDK5 in the brain tissues of normal and scrapie 263K-infected hamsters during incubation period**. A. Western blots. Same amounts of individual brain homogenate were loaded in 12% SDS-PAGE. The scrapie agent 263 K and normal controls were shown on the top of the graphs. Various specific immunoblots were indicated by arrows on the left. B. Quantitative analysis of each gray numerical value of tau, p-tau (Ser396), p-tau (Ser404), p-tau (Ser202/Thr205), GSK3β and CDK5 vs that of β-actin. The average values were calculated from two individual infected hamsters or four individual normal hamsters and presented as mean ± SD. Statistical differences compared with controls were illustrated as *P *< 0.05 and *P *< 0.01. C. Real time PCR analysis of the mRNA transcription of Tau2, Tau4, GSK3β and CDK5. The transcription level of each specific mRNA was determined relative to that of the individual β-actin. The relative intensity of each gene from scrapie-infected hamsters was relative to that of respective gene from normal hamsters that was set to 1. Data are representative of three independent experiments.

In order to investigate the dynamic changes of tau, p-tau (Ser396), p-tau (Ser404), p-tau (Ser202/Thr205), GSK3β and CDK5 in the brains of scrapie-infected hamsters during incubation period, the expressive and transcriptional levels of these biomarkers were evaluated by Western blots and real-time PCR, respectively. Brain samples of the agent 263K-infected hamsters at the 20th, 40th, 50th, 60th and 70th days after inoculation were collected. Western blots showed that the signals of tau, p-tau (Ser202/Thr205), and CDK5 became stronger and that of p-tau (Ser396), p-tau (Ser404) and GSK3β became weaker in the infected specimens compared with the normal controls (Figure 6A). Analysis of the gray value of each sample equilibrated with the individual value of β-actin showed obviously increase of tau, p-tau (Ser202/Thr205) and CDK5 signals, and decrease of p-tau (Ser396), p-tau (Ser404) and GSK3β signals in the infected brain samples (Figure 6B). Real-time PCR tests revealed that alone with the incubation the mRNA levels of Tau4 and CDK5 increased remarkably, whereas that of GSK3β became degressive (Figure 6C). In line with the above observations, the transcripts of Tau2 remained relatively stable. These results indicate that changes of tau profiles and phosphate kinases happen much earlier than the onset of the clinical manifestations, showing a time-relative pattern.

## Discussion

AD, the most common form of dementia, is pathologically characterized by Aβ-amyloid plaques and neurofibrillary tangles [5]. The amyloid cascade hypothesis implies that the extracellular deposition of Aβ is the initial and seminal event of AD pathogenesis and the intraneuronal formation of neurofibrillary changes, composed of hyperphosphorylated tau protein, is a consequence of it. In prion disease, a consistent subset of GSS patients, such as some with the most common P102L PRNP mutation, has been shown to harbor neurofibrillary changes further suggesting that tau pathology is downstream from the extracellular deposition of amyloid, either made up of Aβ or PrP [30]. But this has been partially questioned by the fact that in most cases of human prion diseases, the deposition of abnormal PrP in the brain does not induce a tauopathy.

In this study, Western blot and real-time PCR provide the evidences of higher level of tau protein in the brain homogenates of scrapie-infected hamsters. Increased tau protein probably reflects neuronal, preferentially axonal damage or degeneration. Although the scrapie hamster-adapted agent 263 K and agent 139 A bear distinctly different incubation times [31], the similar increased brain tau at the terminal stages indicates a common phenomenon of scrapie experimental animals, neither related with scrapie strains nor incubation periods.

The phosphorylating status of tau is crucial for its biological activities. Aberrant tau phosphorylation can disrupt axonal transport, which has been proposed as an underlying mechanism possibly giving rise to neurodegeneration [32]. Tau mutants, with serine/threonine targets of GSK3 mutated to glutamate to mimic a permanent state of phosphorylation, are transported at a significantly increased rate compared to wild-type tau. Conversely, tau mutants, in which alanine replaced serine/threonine to mimic permanent dephosphorylation, are transported at a decreased rate compared to wild-type tau [32]. The diverse phosphorylating statuses of tau have been also described in human prion diseases, e.g. increased p-tau in GSS but not in sporadic CJD. Compared with healthy hamsters, inoculations of two scrapie strains into experimental hamsters cause decreased of p-tau at Ser396/Ser404 and increased of p-tau at Ser202/Thr205 in the brains. Tau protein has a series of potential phosphorylating sites, among them only p-tau at Ser396, Ser404 and Ser202/Thr205 has been addressed in this study. One might speculate there are other profiles of p-tau in the brains of human and animal TSEs. However, highly depressed p-tau at Ser396/Ser404 and markedly raised p-tau at Ser202/Thr205 in brains of the hamsters infected by scrapie strongly indicate these changes of p-tau profiles are TSE related events.

CDK5 and GSK3β have been identified as major candidates mediating tau phosphorylation at sites characteristic for neurodegenerative tauopathies. Specific inhibition of GSK3β reduces tau phosphorylation and significantly decreases the overall rate of axonal transport of tau in rat cortical neurons [32]. Alterations of the patterns of other phosphorylation kinase, e.g. CK2 and its subunits, have been also observed in the brain tissues of the experimental and naturally-occurred animal and human TSEs [31]. In this study, we have firstly described that the cerebral level of GSK3β declines and that of CDK5 increases during scrapie pathogenicity. It is reasonable to speculate that the changes of p-tau profiles in the brains of TSEs may link with the alternations of protein kinases.

Our data reveal that the changes of tau profiles and phosphate kinases in scrapie-infected hamsters are time-relative events. In line with the previous observations of PrP^Sc ^deposition and neuropathological abnormality, changes of tau and kinases in brains of scrapie experimental animals appear much earlier than the onset of clinical manifestations. Moreover, gradually declined GSK3β and raised CDK5 levels correspond well with the decrease of p-tau (Ser396 and Ser404) and increase of p-tau (Ser202/Thr205), respectively. These phenomena may highlight the possibility that the increase of p-tau (Ser202/Thr205) is due to the higher level of CDK5 and depression of p-tau (Ser396 and Ser404) owes to the lower level of GSK3β. Additionally, GSK3β and CDK5 seems to show individual tropism on phosphorylating tau, which GSK-3β prefers to act on tau at the C-terminus and CDK5 tends to target the N-terminus [12]. The catalytic characteristics of these two kinases on tau may help to explain the special p-tau profiles in scrapie-infected hamsters. Further immunohistochemistry assays for evaluating the alterations of these biofactors in brains assays for illustrating potential pathological characteristics of tau in scrapie will help to clarify the pathogenesis of prion disease.

## Conclusion

Our results suggest that changes of profiles of p-tau at Ser396, Ser404 and Ser202/Thr205 are illness-correlative phenomena in TSEs, which may arise of the alteration of phosphate kinases. Alterations of tau and kinases in the brains of TSE animals during pathogenicity may consist of a complicate network. It is still unclear when and how these components function and impact each other. Except for the deposits of PrP^Sc ^as the initiative agent, alterations of these bioactive proteins are either intermediate or consequent events in TSE pathogenicity and proposed the potential linkage of these bioactive proteins with the pathogenesis of prion diseases.

## Competing interests

The authors declare that they have no competing interests.

## Authors' contributions

GRW involved in all tests and GRW and SS participated in the acquisition of data and analysis and interpretation of the data. CT, DCF, RMZ and XLL participated in Western blots. CG, BYZ and JH performed the sample preparations. XPD developed the study concept and protocol and all other authors reviewed the protocol and made contributions to study design. GRW and XPD drafted the manuscript and all other authors were involved in revising it critically for important intellectual content and have given final approval of the version submitted.

## Pre-publication history

The pre-publication history for this paper can be accessed here:

http://www.biomedcentral.com/1471-2334/10/86/prepub
